# Socio-demographic characteristics associated with the dietary diversity of Thai community-dwelling older people: results from the national health examination survey

**DOI:** 10.1186/s12889-022-12793-x

**Published:** 2022-02-22

**Authors:** Chalobol Chalermsri, Syed Moshfiqur Rahman, Eva-Charlotte Ekström, Weerasak Muangpaisan, Wichai Aekplakorn, Warapone Satheannopakao, Shirin Ziaei

**Affiliations:** 1 Department of Women’s and Children’s Health, Uppsala University, Uppsala, Sweden; 2 Department of Preventive and Social Medicine, Faculty of Medicine Siriraj Hospital, Mahidol University, Bangkok, Thailand; 3 Department of Community Medicine, Faculty of Medicine Ramathibodi Hospital, Mahidol University, Bangkok, Thailand; 4 Department of Nutrition, Faculty of Public Health, Mahidol University, Bangkok, Thailand

**Keywords:** Dietary diversity, Socio-demographic, Older people, Thailand

## Abstract

**Background:**

Dietary diversity (DD) is an indicator of nutrient intake and is related to health outcomes in older people. Currently, limited research exists regarding factors associated with DD in older people in developing countries, such as Thailand, despite rapid growth in this population. Therefore, this study aims to examine the association between socio-demographic characteristics and DD in Thai older people.

**Methods:**

A cross-sectional study based on the fifth Thai National Health Examination Survey (NHES-V) conducted between 2013 and 2015 was performed. A total of 7,300 nationally representative older participants aged ≥ 60 years were included. The individual-level dietary diversity score (DDS) was assessed as the frequency of consumption of eight food groups using food frequency questionnaires. Each food group was scored from 0 to 4 according to the frequency of consumption. The DDS was calculated as the sum of the scores, ranging from 0 to 32. Socio-demographic characteristics, including age, sex, highest education level, wealth index, living conditions, and residential area, were assessed. Data were analyzed using multiple linear regression and adjusted for complex survey design.

**Results:**

The participants had a mean age of 69.7 (SD 7.6) years. The mean DDS of participants was 18.4 (SD 3.9). In the adjusted model, a higher educational level, a higher wealth index, and living in an urban area were positively associated with DDS, with adjusted β (95% CI) values of 1.37 (1.04, 1.70) for secondary education or higher, 0.81 (0.55, 1.06) for the richest group, and 0.24 (0.10, 0.44) for living in an urban area. Nevertheless, living alone had negative associations with DDS, with a β (95% CI) of - 0.27 (- 0.53, - 0.00).

**Conclusions:**

This study showed that a higher educational level, a higher wealth index, and living in an urban area had a positive association, whereas living alone had a negative association with DD among Thai older participants. Interventions aiming to improve dietary diversity among older people might benefit from targeting more vulnerable groups, particularly those with less education and wealth, those living alone, or those in rural areas.

## Background

The growth of the aging population is one of the major concerns in global health. Globally, the number of people aged 60 years or over surpassed that of under-five children in 2018 [1]. Due to their physical and socio-economic limitations, older people may suffer from several adverse health conditions. The aims of the care process in this population include healthy aging and longevity. Good nutritional status and healthy eating have been shown to prevent mortality from non-communicable diseases, increase quality of life, and enhance healthy aging [2, 3].

Eating a variety of foods is one component of healthy eating and has been recommended in several food-based dietary guidelines (FBDGs) [4, 5]. Dietary diversity (DD) is defined as the number of different food groups consumed over a time period [6] and has been suggested as an indicator for evaluating nutrient intake [7]. DD has been shown to affect gut microbiome, micronutrients level as well as muscle mass, etc. [8–10]. Diversity of food consumption relates to many health outcomes in older people, including malnutrition, cognitive function, and mortality [11–13].

DD is affected by several factors, such as age, sex, physical and mental health, and social and macro-environment factors, such as policies [12, 14–16]. For example, older people tend to have less dietary diversity than the younger population because they rely on their familiar experiences and their dietary restrictions according to their health status [17]. Further, cooking skills have been suggested as a determinant for dietary diversity, and the male elderly who tend to have lower cooking skills are shown to have lower DD than the female elderly [18]. Policies can also influence DD. As an example, a significant improvement was observed in DD among the elderly population in South Africa who were part of a food aid program [19].

Although there are previous studies evaluating factors associated with DD among older people, most of these studies were confined to high-income countries (HICs) in particular regions. Currently, there is limited evidence regarding the factors associated with DD in older people in lower- and middle-income countries (LMICs). There are some differences between high- versus lower- and middle-income countries in factors associated with dietary diversity, such as the equality of education or economic support [20]. Therefore, studies from LMICs are valuable to expand the existing knowledge and enable a comparison between the various settings.

Thailand is an upper-middle-income country in Southeast Asia with one of the most rapidly growing older populations in the world. The number of people aged 60 and over in Thailand is expected to increase from 19.2% to 2019 to 35.6% by 2050 [21]. Currently, the double burden of malnutrition is a common health problem in Thai older people [22]. A previous study of Thai female older people found that economic status acts as a vital determinant of food security [23]. Moreover, in a previous qualitative study, Thai older people emphasized the availability of food in their community as a factor influencing their food choices [17]. However, there is no study evaluating the socio-demographic characteristics associated with DD in older people in Thailand. Considering the importance of dietary patterns for the health and wellbeing of older people, understanding the factors associated with DD is necessary in order to develop effective interventions [24]. Therefore, this study aimed to examine the association between socio-demographic characteristics and DD among older people in Thailand using nationally representative data.

## Methods

### Study design and study population

**Fig. 1:**
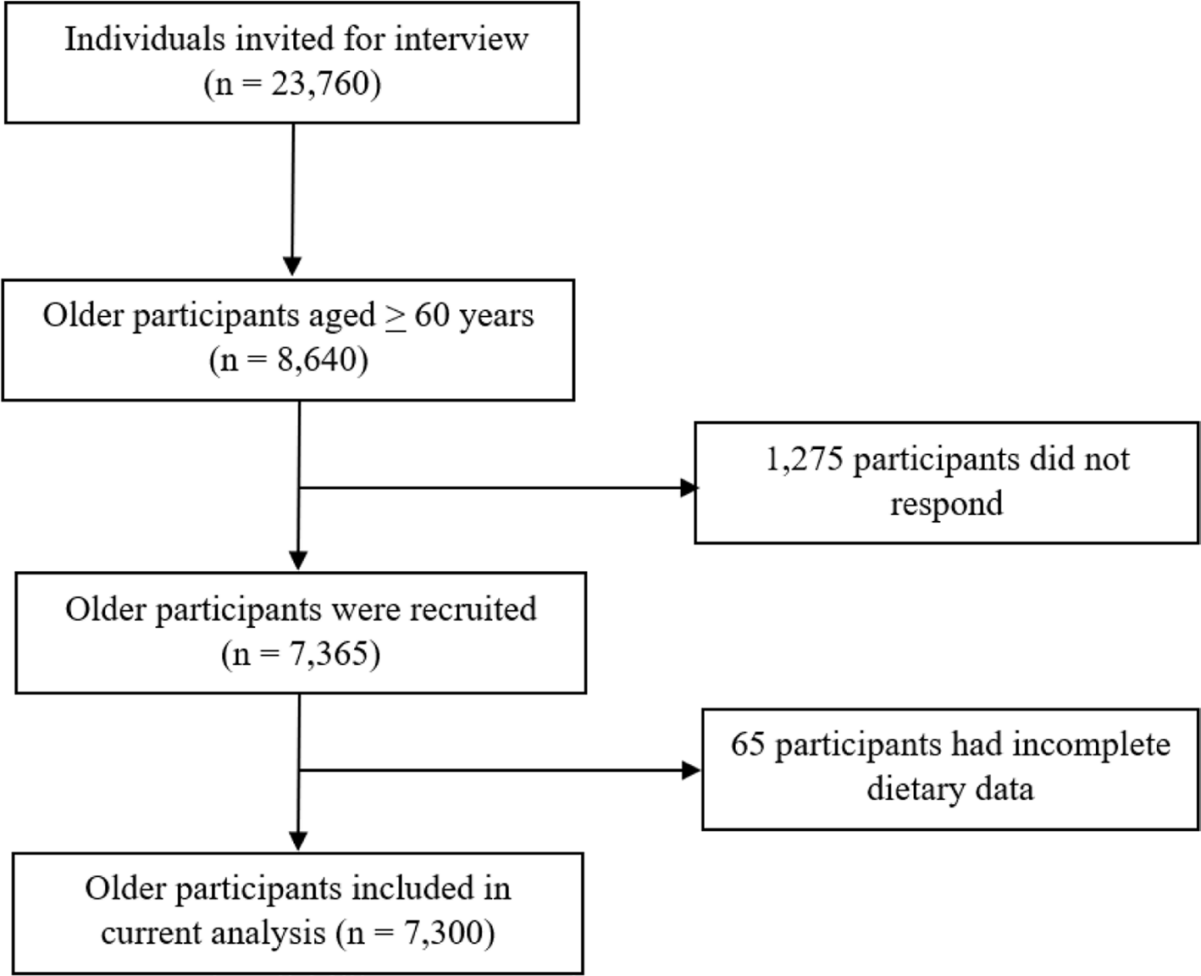
Flow diagram of older participants included in the analysis

This study was a cross-sectional study based on the fifth Thai National Health Examination Survey (NHES-V). The NHES-V was a nationally representative survey using multistage, stratified sampling of the Thai population. The survey was conducted from October 2013 to February 2015, and the demographic characteristics and socio-economic status of the participants were collected via face-to-face interviews. In cases in which older participants could not communicate or had cognitive problems, information was collected from their informant caregivers. The details of this survey are described elsewhere [25]. A total of 23,760 individuals were invited to participate in the NHES-V, among whom 8,640 were aged 60 years and older. The response rate in the older participant group was 85.2%, resulting in 7,365 older participants in this study. Sixty-five participants were excluded from the analysis due to incomplete dietary data. A total of 7,300 older participants were included in the final analysis (Fig. 1). Excluded participants (n = 65) had a significantly higher educational level and wealth index; no other significant differences were observed between the final sample and the excluded participants.

### Data collection

#### Dietary diversity score (DDS)

A 34-item semi-quantitative food frequency questionnaire (FFQ) was used to assess participants’ habitual eating behaviors. The questionnaire was developed based on the commonly consumed food items in Thailand and has been shown to have high internal consistency [26]. FFQs were administered by trained interviewers using pictures of food to facilitate answers.

The dietary diversity score (DDS) was calculated based on a locally adapted version of guidelines, endorsed by the Food and Agriculture Organization of the United Nations (FAO) [27] and an FBDG for Thais [5]. The original version of the DDS from the FAO contained 10 food groups, namely, grain or white roots or tubers, pulse and beans, nuts and seeds, dairy, meat or poultry or fish, eggs, dark green leafy vegetables, other vitamin A-rich fruits and vegetables, other vegetables, and other fruits. In the adapted version, dark green leafy vegetables, other vitamin A-rich fruits and vegetables, other vegetables, and other fruits were collapsed and reclassified as a vegetables group and a fruits group due to the availability of data from the FFQ. Pulse and beans were also merged with the nuts and seeds food group to comply with dietary patterns in the Thai context [5]. The fat and oil group is one of the main food groups in the FBDG for Thailand and an optional food group in the DDS from the FAO [5, 27]. Thus, the oil and fat group was included in this DDS, resulting in 8 food groups. Each food group contained five choices for the frequency of consumption: (1) never eat or eat less than once per month (0 points), (2) eat once to three times per month (1 point), (3) eat once to three times per week (2 points), (4) eat 4 – 6 times per week (3 points), and (5) eat once per day or more (4 points). The DDS was calculated as the sum of the score for each food group, which ranged from 0 to 32. A higher DDS indicates better dietary diversity.

### Socio-demographic characteristics associated with dietary diversity

 The socio-demographic characteristics of the older participants, including sex, age, highest education level, wealth index, living condition and residential area, were collected via face-to-face interviews. In line with previous studies [28], the age of the participants, which was collected from the identification card, was classified as young old (60 – 74 years), old-old (75 – 84 years), or oldest old (≥ 85 years). The highest education level was classified into three categories (no formal education, primary education, and secondary education and above). The wealth index indicated participants’ economic status by assessing participants’ household assets (bed, washing machine, electric water heater, air conditioner, microwave, electric kettle, PC, telephone, car, and flushing toilet). The wealth index was derived from the first factor loading, which was obtained from the principal component analysis. The wealth index score was further categorized into quintiles. The lowest quintile indicated the poorest group, and the highest indicated the richest group [29]. Living condition was dichotomized as living alone or living with others. Residential area was categorized into urban and rural. Urban and rural areas were defined as the areas inside and outside of local municipalities, respectively.

### Data analyses

The data were analyzed by using STATA/SE version 16.1 (StataCorp, College Station, Texas, U.S.A.). Adjustment for complex survey design was applied in the analyses. This adjustment accounted for clustering and weighting of older participants by using a set of Svy commands in STATA [30]. This study used weighting based on the sampling probability against the 2014 registered Thai population [25]. Chi-squared tests were used to compare the background characteristics of participants who were excluded from the analysis with those of the final sample. Descriptive statistics are presented as frequencies and percentages for categorical variables and as the mean with standard deviation (SD) or 95% confidence interval (95% CI) for continuous variables. The normality of the DDS data was examined by constructing a histogram. The mean differences in DDS between two groups and more than two groups were tested by Student’s t-test and one-way analysis of variance (ANOVA), respectively. Factors associated with the DDS were evaluated by using simple and multiple linear regression models after the assumptions for linear regression were met. The association between socio-demographic characteristics and the DDS was represented by *beta* coefficients (β) and 95% CIs. Multicollinearity was also checked with the generalized variance inflation factor (GVIF) [31] using the R statistical software package car [32]. The GVIF did not exceed 1.37 for any variable, indicating no evidence of multicollinearity. All the variables showing a significant association with the DDS in unadjusted models, namely, age, sex, highest educational level, wealth index, living alone, and residential area, were entered in the multivariate analysis. The results were considered statistically significant at a P-value < 0.05.

## Results

A total of 7,300 older participants were included in this study (Fig. 1). The participants had a mean (SD) age of 69.7 (7.6) years. The mean DDS was 18.4 (SD 3.9) (data not shown). Table 1 shows the socio-demographic characteristics of the participants. Approximately 44% of the older participants were male. Nearly 75% of them were 60 – 74 years old. Most of the older participants had completed primary education or higher. The weighted prevalence of living alone was approximately 8%. More than 40% of the older participants lived in urban areas.

**Table 1:** Socio-demographic characteristics of older participants based on the NHES-V, Thailand (n 7,300)

Characteristics	Weighted number (%)
**Male**	3,222 (44.1)
**Age (year)**	
60 – 74	5,380 (73.7)
75 – 84	1,635 (22.4)
≥ 85	285 (3.9)
**Highest educational level**	
No formal education	691 (9.5)
Primary education	5,766 (79.3)
Secondary education or higher	814 (11.2)
**Wealth index**	
Poorest	1,732 (28.9)
Poor	1,061 (17.7)
Average	1,091 (18.2)
Rich	1,121 (18.7)
Richest	989 (16.5)
** Living alone**	576 (7.9)
** Urban**	2,978 (40.8)

*NHES-V* the fifth Thai National Health Examination Survey

^*^Total sample size varies because of the variety of missing values

Table 2 shows the distribution of the mean DDS by the socio-demographic characteristics of the older participants. The mean DDS among the male older participants was slightly higher than among the female older participants (18.4 vs. 18.3, *P*-value 0.007). The mean DDS decreased by age group (*P*-value < 0.001). Older participants who had a higher education level had a significantly higher mean DDS than those who had a lower education level (*P*-value < 0.001). Similarly, the mean DDS of the older participants who had a higher wealth index was higher than that of the poorer group (*P* < 0.001). The mean DDS of the older participants who lived alone was significantly lower than that of the older participants who lived with others (17.9 vs. 18.4, *P*-value <0.001), and the mean DDS of the older participants who resided in an urban area was higher than that of the older participants who lived in a rural area (18.7 vs. 18.1, *P*-value <0.001).

**Table 2:** DDS based on the socio-demographic characteristics of older participants in the NHES-V, Thailand (n 7,300)

Characteristics	Mean (SD)	95% CI	*P*-value*
**Sex**			
Male	18.4 (3.8)	18.3, 18.6	0.007
Female	18.3 (4.0)	18.1, 18.4	
**Age (year)**			
60-74	18.5 (4.0)	18.3, 18.6	<0.001
75-84	18.0 (3.9)	17.9, 18.2	
≥ 85	17.9 (3.6)	17.5, 18.2	
**Highest educational level**			
No formal education	17.3 (4.4)	17.1, 17.5	<0.001
Primary education	18.3 (3.8)	18.2, 18.5	
Secondary education or higher	19.3 (4.4)	19.1, 19.5	
**Wealth index**			
Poorest	17.7 (3.7)	17.5, 18.0	< 0.001
Poor	18.8 (3.8)	18.6, 18.9	
Average	18.2 (3.9)	18.0, 18.4	
Rich	18.5 (3.8)	18.4, 18.7	
Richest	19.0 (4.3)	18.9, 19.2	
**Living condition**			
Living alone	17.9 (4.0)	17.6, 18.1	<0.001
Living with others	18.4 (3.9)	18.3, 18.5	
**Residential area**			
Urban	18.7 (4.4)	18.6, 18.8	<0.001
Rural	18.1 (3.5)	18.0, 18.3	

SD standard deviation, 95% CI 95% confidence interval, *DDS* dietary diversity score, *NHES-V *the fifth Thai National Health Examination Survey

*P-values obtained from Student’s t test or one-way ANOVA and adjusted for complex survey design by Svy commands

Table 3 shows the unadjusted and adjusted linear regression models evaluating the associations between the socio-demographic characteristics and DDS of the older participants. In the unadjusted regression model, female older participants had a lower DDS than male older participants (β - 0.19, 95% CI: - 0.32, - 0.06). Older participants who were older had a significantly lower DDS than the young old group (β - 0.43, 95% CI: - 0.63, - 0.22 for age 75 – 84 years and β - 0.61, 95% CI: - 0.96, - 0.26 for age 85 years or above). Compared to older participants with no formal education, those who had higher education had a significantly higher DDS (β 1.00, 95% CI: 0.77, 1.23 for primary education and β 1.97, 95% CI: 1.70, 2.23 for secondary education and higher). In addition, older participants who were richest had a higher DDS than the poorest groups (β 1.29, 95% CI: 1.05, 1.53). Older participants who lived alone had a significantly lower DDS than those who lived with others (β - 0.51, 95% CI: - 0.75, - 0.27), while older participants who lived in urban areas had a significantly higher DDS than their counterparts who lived in rural areas (β 0.54, 95% CI: 0.39, 0.69).

**Table 3:** Association between the socio-demographic characteristics and DDS of older participants in the NHES-V, Thailand

Characteristics	Unadjusted		Adjusted^a^	
	**β (95%CI)**	***P*** -value**	**β (95%CI)**	***P*** -value**
**Sex**				
Male	Reference		Reference	
Female	-0.19 (-0.32, -0.06)	0.007	0.03 (-0.11, 0.17)	0.671
**Age (year)**				
60 - 74	Reference		Reference	
75- 84	-0.43 (-0.63, -0.22)	<0.001	-0.14 (-0.36, 0.07)	0.177
≥ 85	-0.61 (-0.96, -0.26)	0.002	-0.30 (-0.69, 0.09)	0.119
**Highest educational level**				
No formal education	Reference		Reference	
Primary education	1.00 (0.77, 1.23)	<0.001	0.76 (0.52, 1.03)	<0.001
Secondary education or higher	1.97 (1.70, 2.23)	<0.001	1.37 (1.04, 1.70)	<0.001
**Wealth index**				
Poorest	Reference		Reference	
Poor	1.05 (0.79, 1.32)	<0.001	0.97 (0.70, 1.24)	<0.001
Average	0.47 (0.25, 0.70)	<0.001	0.30 (0.06, 0.54)	0.016
Rich	0.78 (0.56, 1.01)	<0.001	0.56 (0.32, 0.80)	<0.001
Richest	1.29 (1.05, 1.53)	<0.001	0.81 (0.55, 1.06)	<0.001
**Living condition**				
Living with others	Reference		Reference	
Living alone	- 0.51 (- 0.75, - 0.27)	<0.001	- 0.27 (- 0.53, - 0.00)	0.049
**Residential area**				
Rural	Reference		Reference	
Urban	0.54 (0.39, 0.69)	<0.001	0.27 (0.10, 0.44)	0.004

*DDS* dietary diversity score, *NHES-V *the fifth Thai National Health Examination Survey, *β beta* coefficient, *95% CI* *95*% *confidence interval*

^a^Models included age, sex, highest educational level, wealth index, living alone, and residential area

**P-value*s* obtained from linear regression and adjusted for complex survey design by Svy command

In the adjusted models, the results showed that education level, wealth index, and living in an urban area had a positive association, whereas living alone had a negative association with the DDS. Older participants who had a higher education level had a significantly higher DDS than the older participants with no formal education (β 0.76, 95% CI: 0.52, 1.03 for primary education and β 1.37, 95% CI: 1.04 1.70 for secondary and higher education). Similarly, older participants who were richest had a higher DDS than the poorest group (β 0.81, 95% CI: 0.55, 1.06 for the richest group). Regarding living conditions, older participants who were living alone had a lower DDS than participants who were living with others (β - 0.27, 95% CI: - 0.53, - 0.00). Finally, older participants who lived in urban areas had a higher DDS than those who lived in rural areas (β 0.27, 95% CI: 0.10, 0.44). However, sex and age were no longer significantly associated with DDS in the adjusted model.

## Discussion

This study examined the socio-demographic characteristics associated with dietary diversity among community-dwelling older people in Thailand. The results showed that a higher educational level, a higher wealth index, and living in an urban area had a positive association, whereas living alone had a negative association with DD in this population.

In the present study, a higher educational level had a positive association with DD. Older participants educated at the primary school level or above had significantly higher levels of DD than those with no formal education. Additionally, a study in health care units in Brazil showed that older participants who attained education for ≥ 9 years had a higher variety of food group consumption and a higher healthy eating index than those who had 0 – 4 years of education [33] One possible explanation is that education level can influence the level of nutritional knowledge among participants and ultimately result in higher DD. Older participants who attained at least a secondary school education have been shown to have better nutritional knowledge than those with a primary school education [34]. In addition, obtaining nutritional information from various sources has been shown to be more common among older populations with higher education levels [35]. Furthermore, people who have a higher educational level might have higher income and thus better purchasing power than people who have a lower educational level. However, the association between education and DD in older participants is still uncertain. Although many studies have found a positive association between level of education and DD, a negative or no association between education and DD has been reported in some studies [14, 36, 37]. A possible explanation for these results may be differences in the study setting. The study that showed a negative association between education and DD was conducted among Spanish people who were born in the 1940 – 1960 s. The female population in this period had less opportunity to receive education, while they had higher DD than their male counterparts. This might explain the negative association that was found between education and DD in that study [14]. The studies that demonstrated no association between level of education and DD were mainly performed in high-income countries where participants have education equity and obtaining a proper education is common. Therefore, the effect of education on DD might have been diminished. The current study was performed in Thailand, where the education level is heterogeneous across the population; therefore, the association between this factor and DD appeared significant.

With regard to the wealth index, this study found a positive association between the wealth index and DD. Our finding was consistent with a previous study that was conducted among Japanese older participants and found an association between DD and annual income for both sexes [15]. Both individual and household income have been shown to affect elderly participants’ DD [15, 38]. A possible explanation is that participants with better economic status might spend more money on food. A study among Taiwanese older participants found that older participants with higher DD spent two times more money on food purchases than participants with lower DD [39].

In this study, living alone had a negative association with DD. This finding is in agreement with a previous study in Japan that found that older participants who were living alone had significantly lower DD than their counterparts who were living with others [40]. These findings further support the idea that loneliness and social deprivation may affect older people’s eating habits [41]. A qualitative study in the UK found that older participants who were living alone felt that eating alone was less enjoyable than eating with others, and they often purchased less food than when they used to buy food for their whole family [42]. Moreover, a recent qualitative study exploring food choice in Thai older participants showed that participants who were living alone did not feel hungry and often skipped meals as a consequence of loneliness [17].

Another important finding in this study was that older participants who lived in urban areas had higher DD than those who lived in rural areas. This finding is congruent with a previous study in China that showed that urban residents had significantly higher DD than rural residents [43]. Moreover, a nationally representative survey in Korea found that older participants who lived in urban areas had higher healthy eating index scores and DD than their rural counterparts [44]. A possible explanation might be the higher accessibility of food in urban settings. Poor fruit and vegetable consumption in older participants who lived far from supermarkets and stores has been reported previously [45]. The density of food stores and supermarkets in urban areas is significantly higher than that in rural areas [46]. Therefore, older participants in urban areas might be closer to food stores and have more opportunity to access food than those living in rural areas.

In this study, sex did not have an association with DD. This result accords with an earlier study among Brazilian older participants [34]. However, many previous studies have reported a difference in DD based on sex [8, 14]. In addition, no significant association between age and DD was observed in our study. The evidence for the association between age and DD is still inconclusive [14, 38]. A possible explanation for this might be that older participants in the old-old and oldest-old groups (age 75 – 84 and > 85 years) could not perform activities of daily living, such as shopping or preparing food, so their caregivers took responsibility for taking care of and providing food for them [47]. This may be the reason why the association between age and DD was not shown. Another possible explanation is the relatively small sample size, particularly for the oldest-old group, which might have prevented us from detecting the differences between the age groups.

The strengths of this study were the large sample size, relatively high response rate, and use of a nationally representative sample. Moreover, currently, there are few studies on the determinants of DD in LMICs. To the best of our knowledge, no such study has been done in Thailand. Therefore, this study can fill this knowledge gap. In addition, the reliability of the eating habit data could be ensured because the information was derived from older participants or their informant caregivers.

However, this study had several limitations. Due to the cross-sectional nature of the study, the temporal relationship cannot be established. Another concern was that the DDS, because it was derived from a semi-quantitative FFQ, could not reflect the amount of each food group. Moreover, the overall food groups in this study were adjusted to comply with the Thai context and FBDG for Thailand, which makes it difficult to generalize our findings to other settings [5]. Although the FFQs were designed and tested prior to the survey to capture the common dietary pattern in Thailand, there was a possibility of missing some rare food items. Regarding bias, the missing data in assessing the wealth index might cause selection bias. We excluded 65 participants from the final analysis due to incomplete dietary data. The excluded participants had a higher education and wealth index; however, since the number of excluded participants was extremely small (less than 1% of the total sample), the likelihood of selection bias is unlikely in this case. Additionally, the possibility of recall bias should be considered, especially for those with memory problems. Finally, several factors that might have affected DD were not addressed, such as caregivers’ characteristics, or factors regarding food environment such as the food availability, which could lead to residual confounders.

## Conclusions

The study demonstrates the association between socio-demographic characteristics and DD in community-dwelling Thai older participants. A higher education level, a higher wealth index, and living in an urban area had a positive association, while living alone had a negative association with DD in this setting. These findings suggest that policies and nutritional interventions that focus on older people with lower wealth and education levels and older people who live alone or in rural areas might help to increase DD in older people.

## Data Availability

The datasets used or analysed are available from the corresponding author on reasonable request.

## Abbreviations

DD: Dietary diversity
NHES-V: The fifth Thai National Health Examination Survey
DDS: Dietary diversity score
SD: Standard deviation
β: *Beta *coefficient
95% CI: 95% confidence interval
FBDG: Food-Based Dietary Guidelines
HIC: High-income countries
LMIC: Lower- and middle-income countries
FFQ: Food frequency questionnaire
FAO: The Food and Agriculture Organization of the United Nations
ANOVA: Analysis of variance
GVIF: Generalized variance inflation factor
